# Predicting the multi-domain progression of Parkinson’s disease: a Bayesian multivariate generalized linear mixed-effect model

**DOI:** 10.1186/s12874-017-0415-4

**Published:** 2017-09-25

**Authors:** Ming Wang, Zheng Li, Eun Young Lee, Mechelle M. Lewis, Lijun Zhang, Nicholas W. Sterling, Daymond Wagner, Paul Eslinger, Guangwei Du, Xuemei Huang

**Affiliations:** 1 Departments of Public Health Sciences, Pennsylvania State University Hershey Medical Center, Hershey, PA 17033 USA; 2 Department of Neurology, Pennsylvania State University Hershey Medical Center, Hershey, PA 17033 USA; 3 Department of Biochemistry and Molecular Biology, Pennsylvania State University Hershey Medical Center, Hershey, PA 17033 USA; 4 Institute of Personalized Medicine, Pennsylvania State University Hershey Medical Center, Hershey, PA 17033 USA; 5 Departments of Pharmacology, Radiology, Neurosurgery, and Kinesiology, Pennsylvania State University Hershey Medical Center, Hershey, PA 17033 USA

**Keywords:** Parkinson’s disease, Multivariate longitudinal data, Generalized linear mixed-effect model, Dynamic prediction, Motor symptoms, Non-motor symptoms, Imbalance

## Abstract

**Background:**

It is challenging for current statistical models to predict clinical progression of Parkinson’s disease (PD) because of the involvement of multi-domains and longitudinal data.

**Methods:**

Past univariate longitudinal or multivariate analyses from cross-sectional trials have limited power to predict individual outcomes or a single moment. The multivariate generalized linear mixed-effect model (GLMM) under the Bayesian framework was proposed to study multi-domain longitudinal outcomes obtained at baseline, 18-, and 36-month. The outcomes included motor, non-motor, and postural instability scores from the MDS-UPDRS, and demographic and standardized clinical data were utilized as covariates. The dynamic prediction was performed for both internal and external subjects using the samples from the posterior distributions of the parameter estimates and random effects, and also the predictive accuracy was evaluated based on the root of mean square error (RMSE), absolute bias (AB) and the area under the receiver operating characteristic (ROC) curve.

**Results:**

First, our prediction model identified clinical data that were differentially associated with motor, non-motor, and postural stability scores. Second, the predictive accuracy of our model for the training data was assessed, and improved prediction was gained in particularly for non-motor (RMSE and AB: 2.89 and 2.20) compared to univariate analysis (RMSE and AB: 3.04 and 2.35). Third, the individual-level predictions of longitudinal trajectories for the testing data were performed, with ~80% observed values falling within the 95% credible intervals.

**Conclusions:**

Multivariate general mixed models hold promise to predict clinical progression of individual outcomes in PD.

**Trial registration:**

The data was obtained from Dr. Xuemei Huang’s NIH grant R01 NS060722, part of NINDS PD Biomarker Program (PDBP). All data was entered within 24 h of collection to the Data Management Repository (DMR), which is publically available (https://pdbp.ninds.nih.gov/data-management).

## Background

Parkinson’s disease (PD) is an age-related neurodegenerative disorder marked by dopaminergic cell loss in the substantia nigra of the basal ganglia [1, 2]. In the US, nearly half a million people are living with PD and the number is expected to increase over the next decade due to aging alone [3]. Although there are effective medical and surgical procedures to treat the disease, it progresses relentlessly and causes both motor and non-motor dysfunction that lead to significant disability and decreases in quality of life [4–6].

Developing disease-modifying agents to slow, halt, or reverse disease progression has been a focus of PD research. Testing potential neuroprotective agents, however, is hindered by the heterogeneous clinical presentation and progression of PD. A method that can predict the longitudinal trajectories of motor and non-motor symptoms based on information that physicians can obtain easily in a clinical setting will be useful for: 1) stratifying patients according to their progression speed for clinical trials [1], which in turn increases the power of studies; 2) consultation regarding PD prognosis in a clinical setting. Limited work exists, however, for dynamic prediction in PD due to the computational challenge in multivariate set-ups [7].

In the past, mixed-effect models were used commonly for longitudinal data analysis [8–10], however, these were based only on univariate analyses. Extension from a univariate to a multivariate model is straightforward mathematically under the framework of generalized linear mixed effects models (GLMM) to accommodate different types of longitudinal outcomes (continuous or discrete) by adopting different link functions (e.g., identity, logit, or log) [10, 11]. Multivariate GLMM can be fitted for inference simultaneously, incorporating not only the correlation of repeated measures for each outcome within a subject, but also the association of multiple outcomes by utilizing the random effects. This method, however, has limited application for clinical data analysis, particularly in Parkinson’s disease for disease progression prediction. Although the extension is relatively easy, the main practical issue is not trivial and there are several complex layers for the computation due to the numerical integration with respect to the random effects and the increasing dimensionality of parameters involved as more longitudinal outcomes are considered ([7, 12]). Several strategies have been proposed to reduce this burden. For instance, a Bayesian technique based on the Markov Chain Monte Carlo (MCMC) algorithm was applied for parameter estimation and inference instead of the likelihood approach due to its flexibility [13, 14]. In addition, literature exists regarding proposals for reducing the dimensionality of parameters by considering the realization of a single latent process, which is continuous (called a “latent trait”) and represents the unobserved disease severity score that combines information from multivariate longitudinal outcomes [15], and this work has been extended to joint modeling of multivariate longitudinal data and time-to-event outcomes [7]. The limitation of these studies, however, is that the association structure among multiple outcomes is restricted since only a single set of random effects accounts for the interrelationships between them. In addition, some work based on machine learning approaches (i.e., principle component analysis) has emerged for longitudinal data analysis, but has not yet been generalized to multivariate settings with mixed types [12]. Recently, Komárek and Komárková [18] conducted a data-driven clustering analysis based on multivariate continuous and discrete longitudinal data under the Bayesian framework and developed an R package “mixAK” for public usage. To implement multivariate GLMM in predicting chronic disease progression, we adopted this package for analysis because of the reliable and efficient computing performance compared to alternative computing packages/software [16, 17].

To ensure our method applicable to a real clinical setting, we obtained clinical data (Common Data Elements) longitudinally from PD subjects recruited from a tertiary movement disorders clinic [18]. First, we defined three outcome measures for our study: motor, non-motor, and postural instability scores. Second, we established a multivariate GLMM for prediction based on demographic and standardized clinical data. Third, we evaluated our model’s predictive accuracy and further compared its performance with univariate analyses. Lastly, the individual-level prediction of longitudinal trajectories for multiple outcomes was performed. Of note, the prediction of outcomes and their trajectories has not been investigated rigorously to date. These types of analyses may lead to a better understanding of PD heterogeneity and progression. The subjects enrolled in the study were relatively early in their disease (<10 years since their diagnosis), making them an effective cohort for identifying factors that may be amenable to early, effective treatments that may improve clinical management for PD patients. In addition, this work can interrogate questions that are clinically and transnationally important, particularly predicting clinical progression for individual patients.

## Methods

### Clinical and demographic data for PD longitudinal study

A PD cohort with sample size of 76 was followed longitudinally and completed comprehensive study visits at baseline, 18-, and 36-months (Huang R01 NS060722) [19]. PD patients were from a tertiary movement disorders clinic and were free of major or acute medical issues other than PD. The diagnosis of PD was made by movement disorder specialists according to published criteria [20, 21]. Multi-domain clinical scales were obtained at each visit, including the Movement Disorders Society Unified PD Rating Scale (MDS-UPDRS) I-II, Hamilton Depression Rating Scale (HAMD) measuring depression, the Montreal Cognitive Assessment (MoCA) measuring global cognitive function, and the Hoehn and Yahr (HY) Scale. Levodopa equivalent daily dose (LEDD) reflecting dopaminergic drug usage was calculated at each study visit based on published conversion factors [22], and duration of illness (DOI) was defined as the time since PD diagnosis by a medical professional [18]. Besides these clinical measures, important demographic information also was collected at the baseline visit for confounding adjustment. This was a retrospective study, and no power analysis was performed.

### Coding data and defining outcomes for PD longitudinal study

First, the predictor variables including time (months) since baseline visit, age at baseline (years), gender (1 = Male; 0 = Female), education (years), DOI (years), HAMD, MoCA, and LEDD were included to build up a prediction model after controlling for medication quantified by LEDD. Of note is that one point was added to the MoCA raw score if the subject’s education was less than 13 years, and HAMD and LEDD were standardized before analysis [23].

We considered three major clinical outcomes: 1) the MDS-UPDRS-II score that quantified motor aspects of daily living activities using a 5-point scale (0–4). The total sum score from 13 items was treated as a continuous variable with integer scores ranging from 0 (normal) to 52 (the most severe); 2) MDS-UPDRS-I that quantified non-motor aspects of daily living activities. This also was treated as a continuous variable and used the same scale and range of scores as the MDS-UPDRS-II; 3) imbalance, which is known to mark an important functional disability regarding balance and walking. This was a binary outcome with a value of 1 if the HY scale or item 2.12 from the MDS-UPDRS-II (issues with walking and balance) was > = 3, otherwise the term was scored 0. The reason we chose the MDS-UPDRS-I and II subscales as outcome measures is that they are less rater- and drug-state dependent. In particular, the MDS-UPDRS-II was used to assess PD motor symptoms unlike other studies that focused on the MDS-UPDRS-III because of the recent finding that MDS-UPDRS-II was a better predictor for quality of life than the traditional MDS-UPDRS-III motor scale, which depends heavily on the timing of the exam, the medication status of the subject (“on,” “off,” or “transitional”), and the rater [24].

### Statistical methods

Demographic information was summarized using the mean ± SD for continuous variables and the frequency for categorical variables. The normality assumption was investigated for continuous variables (i.e., MDS-UPDRS-I or II) based on graphical checking (e.g., histogram, QQ-plot) and the Anderson-Darling (AD) test. The *p*-values for repeated measures comparisons were obtained from mixed-effect models with random intercepts and a significance level of 0.05.

We randomly split the complete dataset into two sets, the training data and the testing data. Joint modeling of multivariate longitudinal scales was performed using multivariate GLMM because of two main advantages: 1) both continuous and discrete types of outcomes can be analyzed jointly and simultaneously; 2) the correlation among repeated measures and multiple response outcomes can be incorporated into the model. The parameter estimation and inference were obtained from the Bayesian approach by utilizing the package “mixAK” in R software [17]. Then, the individual-level prediction of longitudinal trajectories for each outcome was performed. Note that the prediction methods within the training and testing dataset were different. The details of the statistical model and inference are shown next.

Let *D*_*train*_ and *D*_*test*_ denote a training dataset with sample size *N* and a testing dataset with sample size *N* ^∗^, where *N* may or may not be equal to *N* ^∗^and the two datasets are independent of each other. *D*_*train*_ is used to build the prediction model and *D*_*test*_ is used to evaluate the prediction for new subjects. For observation *i* in *D*_*train*_,we denote *Y*_*ikj*_ as the *j* ^*th*^ measure of the *k* ^*th*^ type of scale for the *i* ^*th*^ subject at time point *t*_*ij*_, which could be continuous or discrete, *i* = 1 , 2 ,  ⋯ *N* ; *k* = 1 , 2 ,  ⋯ *K* ; *j* = 1 , 2 ,  ⋯ *n*_*i*_. Given *Y*_*ikj*_ following a distribution from the exponential family with the dispersion parameter *ϕ*_*k*_, we have the following mean model

$$ g\left(E\left({Y}_{ik j}|{\boldsymbol{X}}_{ik j,}{\boldsymbol{Z}}_{ik j},{\boldsymbol{\gamma}}_{ik}\right)\right)={\boldsymbol{X}}_{ik j}^T{\boldsymbol{\beta}}_k+{\boldsymbol{Z}}_{ik j}^T{\boldsymbol{\gamma}}_{ik} $$

where ***X***_*ikj*_ is the *p*-dimentional covariate for fixed effects with ***β***_*k*_ as the associated *p* × 1 vector of parameters, ***Z***_*ikj*_ is the *q*-dimentional covariate for random effects with ***γ***_*ik*_ as the associated *q* × 1 vector of parameters, *g* is a monotone link function depending on the type of outcomes (e.g., identity function for continuous outcomes, logit function for binary outcomes, and log function for count outcomes), and the random effects ${\boldsymbol{\gamma}}_i={\left({\boldsymbol{\gamma}}_{i1}^T,{\boldsymbol{\gamma}}_{i2}^T,\cdots {\boldsymbol{\gamma}}_{iK}^T\right)}^T\sim MVN\left(\boldsymbol{\mu}, \Sigma \right)$. Note that Σ not only takes into account the correlation of repeated measures of each outcome, but also incorporates the association between multiple outcomes. In addition, this is a hierarchically centered GLMM [25], thus ***X***_*ikj*_ and ***Z***_*ikj*_ may not contain the same variables due to the identifiability problem. Of note, ***Y***_*i*_ ⊥ ***Y***_*j*_ , ***γ***_*i*_ ⊥ ***γ***_*j*_ for *i* ≠ *j* and given ***γ***_*ik*_,the random variables ${Y}_{ik1},{Y}_{ik2},\cdots {Y}_{ik{n}_i}$ are independent of each other for the *i* ^*th*^ subject. Given that the parameter vector is $\Theta ={\left\{{\boldsymbol{\beta}}_k^T,\boldsymbol{\mu}, \varSigma, {\phi}_k\right\}}_{k=1}^K$, the likelihood is shown as below

$$ L\left(\Theta; \mathbf{Y},\mathbf{X},\mathbf{Z}\right)=\prod_{i=1}^N\int \prod_{k=1}^K\prod_{j=1}^{n_i}f\left({Y}_{ik j}|{\boldsymbol{\beta}}_k,{\phi}_k,{\boldsymbol{\gamma}}_{ik}\right)f\left({\boldsymbol{\gamma}}_i|\boldsymbol{\mu}, \Sigma \right)d{\boldsymbol{\gamma}}_i $$

Here, we utilize the Bayesian approach based on MCMC for parameter estimation, inference, and prediction. The vague priors are used for all elements in Θ, and *M* (i.e., *M* = 2000 after burn in) posterior samples are obtained for the parameters and random effects denoted by $\left\{{\Theta}^{(m)},{\boldsymbol{\gamma}}_{ik}^{(m)},m=1,2,\cdots M\right\}.$The procedures for prediction are performed as follows:

I. Internal prediction for PD subjects using the training dataset

For the *l* ^*th*^ PD subject in the training set *D*_*train*_ (*l* = 1, 2, ⋯*N*), we aim to predict the measures at a future time point *t* ^′^ given the outcome history ${\mathcal{Y}}_l\left({t}^{\prime}\right)=\left\{{Y}_{lkj},0\le {t}_{ij}<{t}^{\prime}\right\}$ and the covariate history ${\mathcal{X}}_l\left({t}^{\prime}\right)=${***X***_*lkj*_ , ***Z***_*lkj*_ , 0 ≤ *t*_*ij*_ ≤ *t* ^′^}. The prediction can be achieved by plugging in the estimates of the parameters and random effects from the posterior samples. For a continuous outcome, the predicted value based on the *m* ^*th*^ posterior sample is

$$ {Y}_{lk}^{(m)}\left({t}^{\hbox{'}}\right)={\boldsymbol{X}}_{ik}^T\left({t}^{\hbox{'}}\right){\boldsymbol{\beta}}_k^{(m)}+{\boldsymbol{Z}}_{ik}^T\left({t}^{\hbox{'}}\right){\boldsymbol{\gamma}}_{ik}^{(m)}+{\varepsilon}_{ik}^{(m)}\left({t}^{\hbox{'}}\right) $$

where ${\varepsilon}_{ik}^{(m)}\left({t}^{\prime}\right)\sim N\left(0,{\sigma}_{\varepsilon}^{2(m)}\right)$. Thus, the predicted estimate is shown as below

$$ {\widehat{Y}}_{ik}\left({t}^{\hbox{'}}\right)=\frac{1}{M}{\sum}_{m=1}^M{Y}_{lk}^{(m)}\left({t}^{\hbox{'}}\right) $$

Similar procedures can be followed for the other types of outcomes.

II. External prediction of PD subjects using the testing dataset

For the *l* ^*th*^ new PD subject in the testing dataset *D*_*test*_ (*l* = 1, 2, ⋯*N* ^∗^), we also aim to predict the measures at a future time point *t* ^′^ given the outcome history ${\mathcal{Y}}_l\left({t}^{\prime}\right)$ and the covariate history ${\mathcal{X}}_l\left({t}^{\prime}\right)$ defined the same as above, and the expectation can be calculated with respect to the posterior distribution of the parameters *f*(Θ| *D*_*train*_). The key issue is to obtain the estimate of random effects [26] as shown below

$$ f\left({\boldsymbol{\gamma}}_l|{\mathcal{Y}}_l\left({t}^{\hbox{'}}\right),\Theta \right)\propto f\left({\mathcal{Y}}_l\left({t}^{\hbox{'}}\right)|{\boldsymbol{\gamma}}_l,\Theta \right)f\left({\boldsymbol{\gamma}}_l|\Theta \right) $$

The sample of random effects can be drawn from the above posterior distribution after replacing Θ by Θ^(*m*)^, denoted by ${\boldsymbol{\gamma}}_{\boldsymbol{l}}^{(m)}$for *m* = 1 , 2 ,  ⋯ *M*. The procedures to obtain the predictive measure ${\widehat{\ Y}}_{ik}\left({t}^{\prime}\right)$will be the same as above.

In addition, univariate longitudinal analyses were conducted with a different specification in Σ, where the correlation among outcomes was assumed to be 0.

Finally, we evaluated the predictive accuracy for continuous outcomes by using the statistical criteria, the root mean square error (RMSE) and the absolute bias (AB), with less values indicating better goodness-of-fit [27, 28] and defined by

$$ {RMSE}_k=\sqrt{\frac{\sum_{i=1}^N{\sum}_{j=1}^{n_i}{\left[{Y}_{ikj}-E\left({Y}_{ikj}|{\boldsymbol{X}}_{ikj},{\boldsymbol{Z}}_{lkj}\right)\right]}^2}{\sum_{i=1}^N{n}_i}} $$

$$ {AB}_k=\frac{\sum_{i=1}^N{\sum}_{j=1}^{n_i}\mid {Y}_{ikj}-E\left({Y}_{ikj}|{\boldsymbol{X}}_{ikj},{\boldsymbol{Z}}_{lkj}\right)\mid }{\sum_{i=1}^N{n}_i} $$

where *N* is the sample size, and *n*_*i*_ is the number of repeated observations for the *i* ^*th*^ subject. With regards to binary outcomes, we evaluated the predictive accuracy by assessing the discrimination ability using the receiver operating characteristic (ROC) curve with varied threshold on the predictive probabilities. The area under the curve (AUC) can be calculated, and the comparison between the AUCs can be tested based on the DeLong’s method [29].

## Results

### Multivariate GLMM under Bayesian framework for PD longitudinal study

To evaluate the prediction ability for internal (existing) and external (new) subjects, we randomly selected the majority of PD patients (*N* = 70) as the training data to develop the prediction model and avoid convergence issues, and the remaining PD patients (*N* = 6) as the testing data due to the relatively small overall sample size. Provided that three outcomes for the *i* ^*th*^ subject at the *j* ^*th*^ time visit, MDS-UPDRS-II (denoted by *Y*_*i*1*j*_), MDS-UPDRS-I (denoted by *Y*_*i*2*j*_), and imbalance status (denoted by *Y*_*i*3*j*_), the multiva:

$$ E\left({Y}_{i1j}|{\gamma}_{i1}\right)={\beta}_{10}+{\beta}_{11} time+{\beta}_{12} Agebaseline+{\beta}_{13} Gender+{\beta}_{14} Education+{\beta}_{15} DOI+{\beta}_{16} HAMD+{\beta}_{17} MoCA+{\beta}_{18} LEDD+{\gamma}_{i1} $$

$$ E\left({Y}_{i2j}|{\gamma}_{i2}\right)={\beta}_{20}+{\beta}_{21} time+{\beta}_{22} Agebaseline+{\beta}_{23} Gender+{\beta}_{24} Education+{\beta}_{25} DOI+{\beta}_{26} HAMD+{\beta}_{27} MoCA+{\beta}_{28} LEDD+{\gamma}_{i2} $$

$$ logit\left(\Pr \left({Y}_{i3j}=1|{\gamma}_{i3}\right)\right)={\beta}_{30}+{\beta}_{31} time+{\beta}_{32} Agebaseline+{\beta}_{33} Gender+{\beta}_{34} Education+{\beta}_{35} DOI+{\beta}_{36} HAMD+{\beta}_{37} MoCA+{\beta}_{38} LEDD+{\gamma}_{i3} $$

where only random intercepts were considered and the mechanism of missing at random is assumed. We ran the MCMC algorithm for 4000 burn-in and 2000 subsequent iterations with 1:10 thinning to get samples from the joint posterior distribution (two sampled chains with different sets of initial values using the function “GLMM_MCMC”). Under default options in the R package of “mixAK,” the shift vector and the scale matrix were pre-specified. The prior distributions for the shifted-scaled means and the *β* parameters were normal distributions, the inverse of the covariance matrix followed a Wishart distribution, and the dispersion parameter *ϕ* was a gamma distribution, which are non-informative.

### Baseline and longitudinal demographic and clinical data

**Fig. 1:**
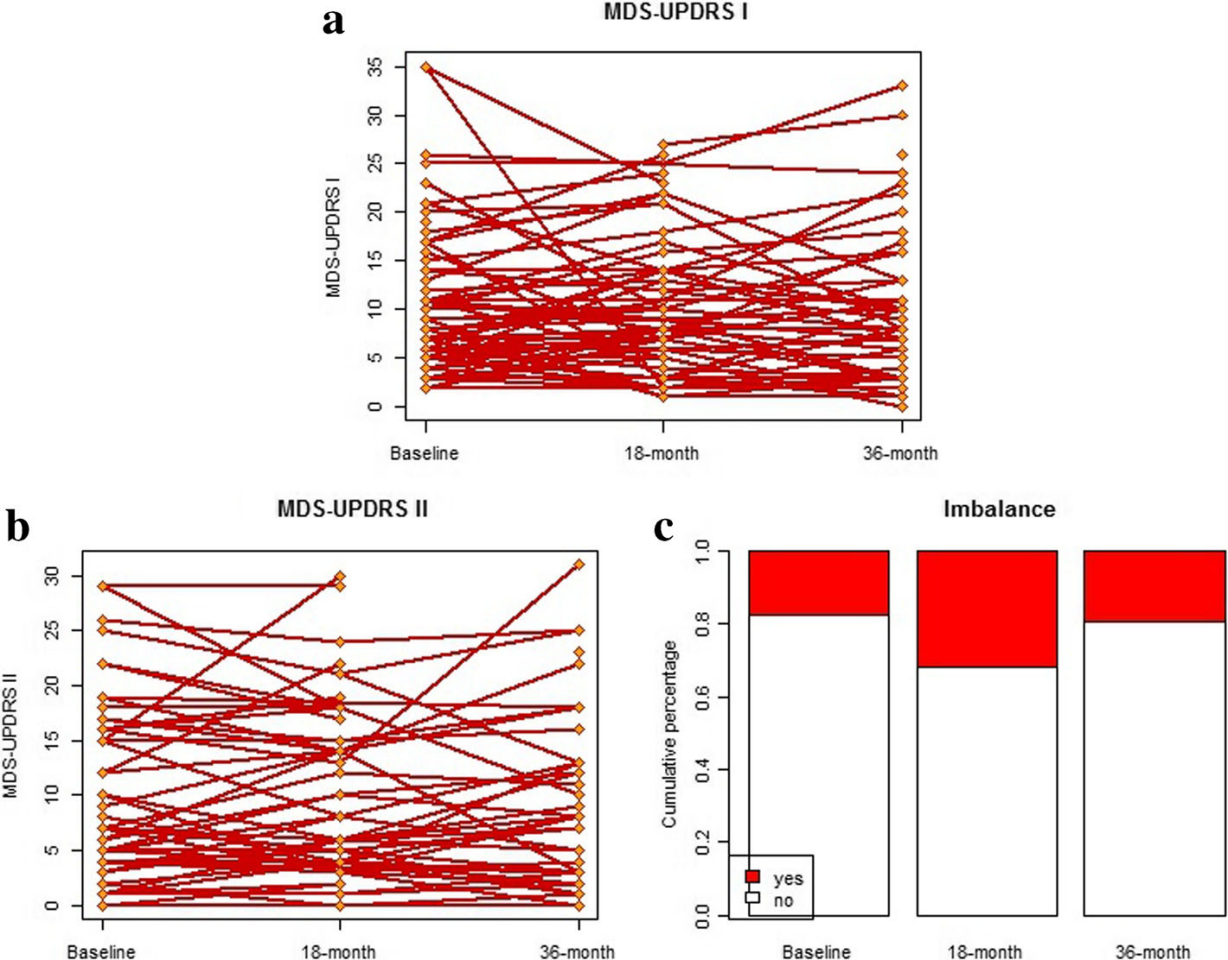
Plots for PD subjects on the three outcome measures over time. For the spaghetti plots of MDS-UPDRS subscales I (**a**) and II (**b**), each solid line in red represents each PD subject and the dots show the measurement at each visit. For the Box plots of imbalance (**c**), each column corresponds to each visit, and the two areas (white and red) within each of the columns correspond to the proportion of the corresponding imbalance categories (Yes/No)

The summary results for all PD subjects are provided in Table 1. For the overall PD cohort, the mean ± SD for age at baseline and education years were 63.2 ± 8.4 and 14.9 ± 2.7 years, respectively. Subjects were 61.8% male, although only 57.4% of the males remained at the 36-month follow-up visit. There was less attrition for females, whose rate of return at the 36-month visit was 86.2%. At baseline, the mean ± SD disease duration was 5.1 ± 5.5 years (range 0.1–25.3 years). Two subjects had missing values for item 2.12 on the MDS-UPDRS-I subscale at baseline. Of the remaining 74 subjects, 4 had balance problems at baseline (item 2.12 ≥ 3), 5 had balance problems at the 18-month visit, and 7 did at the 36-month visit. There was a significant trend for MoCA scores to decrease (*p*-values = 0.03), along with a significant increase in LEDD values (p-value < 0.001), over the 36-month study period. The MDS-UPDRS-I and II scores were not significantly different at the three study visits. Imbalance, however, had a significant trend of improvement over time, with the highest rate at the 18-month visit that then decreased at the 36-month visit, possibly due to informative dropouts (e.g., people who have balance issues may have a harder time continuing in the study) or inaccuracy of coding (see more discussion in the limitation section). The subject-level trajectories of the MDS-UPDRS-I and II (treated as continuous variables), and the percentages of imbalance over time (binary variable), were shown in Fig. 1. The normality assumptions were satisfied with p-values = 0.07 for MDS-UPDRS-I and 0.18 for MDS-UPDRS-II based on the AD tests. Of note is that there was substantial heterogeneity across PD subjects and thus mixed-effect models with subject-level random effects (e.g., a random intercept) were adopted for model fitting.

**Table 1:** Data summary for PD subjects across time. Note that the *p*-values for repeated measures were obtained from the mixed-effect models with random intercepts

	Baseline (*N* = 76)	18-month (*N* = 65)	36-month (*N* = 52)	*P*-value
Age at baseline (years)	63.2 ± 8.4	NA	NA	NA
Education (years)	14.9 ± 2.7	NA	NA	NA
Gender, Male: Female	47:29	NA	NA	NA
DOI (years)	5.1 ± 5.5	NA	NA	NA
HAMD	7.7 ± 4.6	7.4 ± 4.5	6.8 ± 6.4	0.35
MoCA	24.9 ± 3.6	24.7 ± 4.2	24.3 ± 4.1	0.03
LEDD	608 ± 465	823 ± 547	917 ± 578	<0.001
H&Y Scale, 0:1:2:3:4:5	2:29:33:12:0:0	0:14:29:14:5:1	2:7:34:8:1:0	0.66
MDS-UPDRS				
Part I	10.9 ± 7.7	10.2 ± 7.0	10.2 ± 8.1	0.56
Part II	9.0 ± 7.5	9.4 ± 7.6	9.0 ± 8.1	0.27

### Impact of demographic and clinical data on outcome measures

The estimation results were summarized by the median and 95% credible intervals from the posterior distribution based on the multivariate GLMM under the Bayesian framework, which are shown in Table 2. The HAMD and DOI were associated positively with the MDS-UPDRS-II motor scores of daily living, whereas MoCA scores were associated negatively. Higher HAMD scores were associated positively with MDS-UPDRS-I, but education was associated negatively with this outcome measure of the non-motor aspects of daily living. Also, there was a significant temporal trend for the risk of imbalance that was consistent with the initial analysis of the data (Table 1). MoCA scores also were associated significantly and negatively with imbalance. Higher LEDD values appeared to significantly improve imbalance but did not affect MDS-UPDRS-I or II subscale scores. Interestingly, there also was a gender difference regarding imbalance, where male subjects were less likely to have imbalance problems. We also investigated the associations between MoCA and various factors including age at baseline, education, gender, DOI, HAMD, and LEDD based on a linear mixed-effect model and found no significant effects except for age at baseline and gender. This implies that the significant effect of MoCA on the outcome measures was not due to collinearity or confounding issues from the other factors (i.e., education, gender, DOI, HAMD, and LEDD). Using the multivariate GLMM method, the correlation estimate between the MDS-UPDRS-II and I was 0.55, which seemed smaller than the empirical estimate of 0.66 reported by He et al. [11], and this could be due to the fact that we adjusted for potential confounding variables in our current model, whereas He et al. did not.

**Table 2:** Results from the multivariate generalized linear mixed modeling under the Bayesian framework. The data represent the median and 95% credible intervals for the outcome measures MDS-UPDRS-II, MDS-UPDRS-I, and Imbalance

	Median	2.50%	97.50%
MDS-UPDRS-II (motor)			
Intercept	13.522	0.076	25.197
Time	−0.042	−0.095	0.011
Age at Baseline	0.035	−0.147	0.211
Education	−0.108	−0.604	0.419
Gender (=Male)	0.805	−2.381	3.904
DOI^*^	**0.549**	**0.169**	**0.919**
HAMD^*^	**0.283**	**0.087**	**0.492**
MoCA^*^	**−0.320**	**−0.584**	**−0.052**
LEDD	1.066	−0.570	2.612
MDS-UPDRS-I (non-motor)			
Intercept	26.351	10.126	38.947
Time	−0.016	−0.075	0.046
Age at Baseline	0.075	−0.098	0.250
Education^*^	**−0.575**	**−1.075**	**−0.107**
Gender (=Male)	−1.925	−5.203	1.036
DOI	0.037	−0.306	0.397
HAMD^*^	**0.439**	**0.217**	**0.693**
MoCA	−0.311	−0.609	0.008
LEDD	0.360	−1.250	1.993
Imbalance			
Intercept	6.096	−6.903	23.289
Time^*^	**−0.109**	**−0.579**	**−0.001**
Age at Baseline	0.181	−0.021	0.853
Education	−0.148	−1.184	0.823
Gender (=Male)^*^	**−4.657**	**−21.182**	**−0.790**
DOI	0.312	−0.027	2.233
HAMD	0.023	−0.302	0.541
MoCA^*^	**−0.589**	**−2.336**	**−0.164**
LEDD^*^	**2.827**	**0.675**	**11.893**

Abbreviations: *MDS-UPDRS* the Movement Disorder Society-sponsored Unified Parkinson’s Disease Rating Scale, *DOI* duration of illness, *HAMD* Hamilton Depression Rating Scale, *MoCA* the Montreal Cognitive Assessment, *LEDD* Levodopa equivalent daily dose

*95% credible interval does not include 0, indicating the signifiance

### Prediction accuracy of the GLMM and comparison with univariate models

The predictive accuracy, RMSE and AB, were 2.01 and 1.53 for the MDS-UPDRS-II and 2.89 and 2.20 for the MDS-UPDRS-I, respectively, and also ROC-AUC was 0.992 for the imbalance outcome, using the GLMM under the Bayesian framework. For comparison, similar strategies were applied for the data but assuming zero correlations among three types of outcomes, and the RMSE and AB for the MDS-UPDRS-II were 1.98 and 1.52, and 3.04 and 2.35 for the MDS-UPDRS-I, and also ROC-AUC was 0.996 (*p* = 0.29). It showed that the multivariate GLMM had improved performance in prediction particularly for MDS-UPDRS-I compared to univariate models; however, a larger trial or cohort study is needed for further validation.

### Individual-level predictions based on the multivariate GLMM

**Fig. 2:**
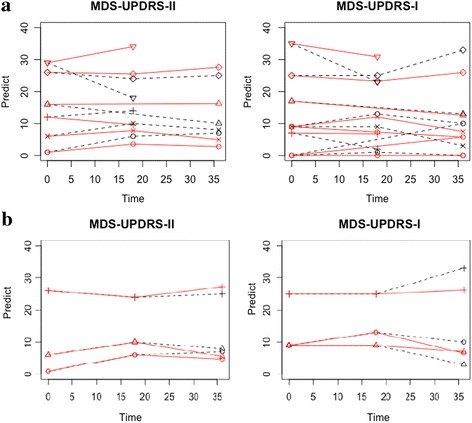
Plots for precition results for all subjects in the testing data. The above two figures (**a**) are for the prediction of 18-month and 36-month mesuares based on baseline data only; the bottom two figures (**b**) are for the prediction of 36-month measures based on both of baseline and 18-month data

Lastly, we conducted subject-level predictions based on our multivariate GLMM for the testing data that included 6 subjects. We conducted prediction of each measure for each subject at the 18-month and 36-month visits shown in Fig. 2, and the majority (~80%) of the 95% credible intervals included the true observed values. Table 3 shows the results for one PD subject randomly selected from the testing cohort including the prediction at 18- and 36-month visits using only baseline data, and also at the 36-month visit alone by incorporating both baseline and18-month data. As expected, the inclusion of additional information (e.g., both the baseline and 18-month visits) improved the prediction (less bias) and decreased the 95% credible intervals.

**Table 3:** The prediction results for one PD subject. Test data represent the predicted values for the outcome variables, with 95% credible intervals in parentheses

	18-month	36-month
MDS-UPDRS-II (motor)		
True value from subject	24	25
Based on Baseline^a^	25.53 (20.08, 30.9)	27.63 (22.30, 33.10)
Based on Baseline and 18-month^b^		27.17 (23.18, 31.17)
MDS-UPDRS-I (non-motor)		
True value from subject	25	33
Based on Baseline^a^	23.14 (17.47, 28.75)	25.86 (20.04, 31.76)
Based on Baseline and 18-month^b^		26.16 (21.32, 30.95)
Imbalance		
True value	1	0
Based on Baseline^a^	1.00 (0.52, 1.00)	1.00 (0.35, 1.00)
Based on Baseline and 18-month^b^		1.00 (0.34, 1.00)

^a^ Each prediction for the 36-month outcome measure was based on baseline data alone

^b^ a combination of the baseline and 18-month data

## Discussion

Similar to many chronic diseases, PD presents with heterogeneous symptoms that may coexist and progress at different rates. Thus, identifying factors to predict disease progression in multiple domains for this population is challenging due to the limited number of longitudinal studies using uniform data collection procedures and few advanced statistical approaches. In the current study, we sought to identify critical factors associated with PD progression while accounting for the heterogeneous and longitudinal nature of motor and non-motor symptoms by using NIH Common Data Elements (Table 1) developed for PD research. We then adopted a joint multivariate GLMM to establish a prediction model for assessing the longitudinal progression of motor and non-motor aspects of daily living activities and balance.

The present results demonstrated that the HAMD and DOI significantly impacted MDS-UPDRS-II subscale scores, with higher HAMD scores and increased DOI leading to higher MDS-UPDRS-II scores. MoCA had a significant negative effect on this outcome measure, indicating that deteriorating cognitive function resulted in increased difficulties with motor aspects of daily living activities. The HAMD and education significantly affected MDS-UPDRS-I subscale scores such that subjects with more severe depression and less education were more likely to report increased difficulties with non-motor aspects of daily living activities. The current findings make intuitive sense in a clinical setting and are consistent with previous literature reporting that disease duration, severe depressive symptoms, and cognitive impairment negatively impact PD patients overall ([30–33]). For example, depression previously was reported as a significant predictor of non-motor aspects of daily living activities [32]. In addition, DOI was a significant predictor of motor aspects of daily living activities (i.e., MDS-UPDRS-II) [31].

The negative association of total MoCA scores not only with motor symptoms (MDS-UPDRS-II) but also imbalance is intriguing. In the past, cognitive functions (performance on frontal-executive tasks or global cognitive functions assessed by total MMSE or MoCA scores) were reported to be correlated with symptoms assessed by MDS-UPDRS total scores or sub-scores (i.e., bradykinesia or postural instability) ([33–36]). These past studies, however, were based mainly on simple correlation analyses using cross-sectional data with limited variables of interest. The current finding of a significant association between global cognitive function and MDS-UPDRS-II and imbalance using longitudinal multivariate modeling confirms the importance of cognitive ability in quality of life and clinically-important outcomes. Most importantly, the differential contribution of the common clinical variables (DOI, depression, and MoCA) on MDS-UPDRS-II and I provides a foundation for models to predict the individual trajectory of motor and/or non-motor symptoms.

In our study, we established a prediction model of PD progression based on multivariate longitudinal outcomes by considering important demographic and clinical risk factors, and conducted both internal (the training dataset) and external (the testing dataset) predictions for evaluation. As shown in Fig. 2 and Table 3, the model led to a well-accepted prediction for external individual patients with the majority (~80%) of predictive credible intervals including the observed values. In addition, the bias for prediction of the MDS-UPDRS-II is small; however, the prediction bias at the 36-month visit for the MDS-UPDRS-I is relatively large but tends to get smaller when more information is incorporated (i.e., a combination of the baseline and 18-month data). It is interesting to note that the variability for the MDS-UPDRS-I is larger than that for the MDS-UPDRS-II (i.e., the mean of individual-level standard deviations for MDS-UPDRS-I and II: 3.61 and 2.78) since the MDS-UPDRS-I is a composite score of multiple non-motor symptoms that are more subjective. The current approach introducing a Bayesian framework gains more power for prediction by incorporating the correlations not only among repeated measures, but also among multiple outcomes. Demonstrating the ability of our model to predict these outcomes simultaneously is a novel finding. Compared to univariate longitudinal analysis, our method achieved non-inferior predictive accuracy. In particular, borrowing information from motor symptoms and imbalance can improve the prediction of non-motor symptom progression from using only a subject’s own history/observed data.

This work has several limitations: 1) at the time of the current data analysis, the data collection time period was up to 36-months and included only three visits, which may have contributed to the lack of a clear, significant, temporal trend for clinical progression. A longitudinal cohort with well-characterized clinical features and measures of progression over a longer follow-up time-frame is important for us to build a prediction model that will increase in accuracy. 2) The drop out rate was >20% from baseline to the 36-month visit, and preferentially male. The differential drop out may influence our results and ability to predict individual outcomes. In addition, although postural instability is a major clinical feature, our outcome measurement consisted only of very limited information from a single source (MDS-UPDRS-II, item 2.12). This limitation may contribute to the insensible finding of “improved balance” over time, and low prediction accuracy in this domain. 3) Identifying the baseline risk factors that can predict PD progression continues to be a work in progress. For the current study, we used some of the features collected under the NIH CDE for PD. Future models should integrate new information as state-of-the-art biomarker discoveries yield more information (e.g., from fluid and imaging research). 4) The linear temporal trend commonly is considered for the current analysis (see the fitted models above), but this would be less plausible in practice. A flexible strategy can be applied to relax it by adopting a spline regression in a semi-parametric framework but at the expense of more computation load.

## Conclusions

In the current study, we applied multivariate generalized linear mixed effect models that incorporated not only the correlation among repeated measurements but also the correlations among multiple outcome variables to predict disease progression. This is the first study to conduct a prediction study of PD progression by considering multiple, longitudinal, and clinically meaningful outcomes (i.e., MDS-UPDRS subscales II, I, and imbalance). Using the real clinical data collected via NIH CDEs, the predictive accuracy of joint multivariate modeling was evaluated and compared to univariate modeling. The non-inferiority of the joint multivariate modeling with regards to bias and RMSE compared to univariate modeling demonstrated the promise of the proposed model for predicting PD progression in a clinical setting and/or for subject selection into clinical trials.

## Abbreviations

DOI: Duration of illness
HAM-D: Hamilton Depression Rating Scale
HY Scale: Hoehn and Yahr Scale
LEDD: Levodopa equivalent daily dose
MDS-UPDRS: the Movement Disorder Society-sponsored Unified Parkinson’s Disease Rating Scale
MoCA: the Montreal Cognitive Assessment
PD: Parkinson’s disease
PDBP: Parkinson’s disease Biomarker Program

## References

[1] Goetz CG, Tilley BC, Shaftman SR, Stebbins GT, Fahn S, Martinez-Martin P, Poewe W, Sampaio C, Stern MB, Dodel R, Dubois B, Holloway R, Jankovic J, Kulisevsky J, Lang AE, Lees A, Leurgans S, LeWitt PA, Nyenhuis D, Olanow CW, Rascol O, Schrag A, Teresi JA, van Hilten JJ, LaPelle N, Movement Disorder Society UPDRS Revision Task Force (2008). Movement Disorder Society-sponsored revision of the unified Parkinson's disease rating scale (MDS-UPDRS): scale presentation and clinimetric testing results. Mov Disord.

[2] Lim SY, Fox SH, Lang AE (2009). Overview of the Extranigral aspects of Parkinson disease. Arch Neurol.

[3] Rodriguez M, Rodriguez-Sabate C, Morales I, Sanchez A, Sabate M (2015). Parkinson's Disease as a result of aging. Aging Cell.

[4] Lewis SJ, Dove A, Robbins TW, Barker RA, Owen AM (2003). Cognitive impairments in early Parkinson's disease are accompanied by reductions in activity in frontostriatal neural circuitry. J Neurosci.

[5] Muslimovic D, Post B, Speelman JD, Schmand B (2005). Cognitive profile of patients with newly diagnosed Parkinson disease. Neurology.

[6] Kehagia AA, Barker RA, Robbins TW (2010). Neuropsychological and clinical heterogeneity of cognitive impairment and dementia in patients with Parkinson's disease. Lancet Neurol.

[7] Wang J, Luo S, Li L (2016) Dynamic prediction for multiple repeated measures and event time data: an application to Parkinson's disease. 10.1214/17-AOAS1059 PMC5656296 29081873

[8] Diggle P, Diggle P (2002) Analysis of longitudinal data. Oxford; New York: Oxford university press.

[9] Laird NM, Ware JH (1982). Random-effects models for longitudinal data. Biometrics.

[10] McCulloch CE, Searle SR, Neuhaus JM (2008). Generalized, linear, and mixed models.

[11] Johnson RA, Wichern DW (2007). Applied multivariate statistical analysis.

[12] Yan F, Lin, X, Huang, X. (2017) Dynamic prediction of disease progression for leukemia patients by functional principal component analysis of longitudinal expression levels of an oncogene. Annals of Applied Statistics; In press.

[13] Berchialla P, Baldi I, Notaro V, Barone-Monfrin S, Bassi F, Gregori D (2009). Flexibility of Bayesian generalized linear mixed models for oral health research. Stat Med.

[14] Gilks WR, Richardson S, Spiegelhalter DJ (1996). Markov chain Monte Carlo in practice.

[15] Dunson DB (2003). Dynamic latent trait models for multidimensional longitudinal data. J Am Stat Assoc.

[16] Komárek A (2009). A new R package for Bayesian estimation of multivariate normal mixtures allowing for selection of the number of components and interval-censored data. Computational Statistics & Data Analysis.

[17] Komárek A, Komárková L (2014). Capabilities of R package mixAK for clustering based on multivariate continuous and discrete longitudinal data. J Stat Softw.

[18] Sterling NW, Wang M, Zhang L, Lee EY, Du G, Lewis MM, Styner M, Huang X (2016). Stage-dependent loss of cortical gyrification as Parkinson disease "unfolds". Neurology.

[19] Lewis MM, Du G, Lee EY, Nasralah Z, Sterlin NW, Zhang L, Wagner D, Kong L, Troster AI, Styner M, Eslinger PJ, Mailman RB, Huang X (2016). The pattern of gray matter atrophy in Parkinson's disease differs in cortical and subcortical regions. J Neurol.

[20] Hughes AJ, Ben-Shlomo Y, Daniel SE, Lees AJ (1992). What features improve the accuracy of clinical diagnosis in Parkinson's disease: a clinicopathologic study. Neurology.

[21] Hughes TM, Rosano C, Evans RW, Kuller LH (2013). Brain cholesterol metabolism, oxysterols, and dementia. J Alzheimers Dis.

[22] Tomlinson CL, Stowe R, Patel S, Rick C, Gray R, Clarke CE (2010). Systematic review of levodopa dose equivalency reporting in Parkinson's disease. Mov Disord.

[23] Nasreddine ZS, Phillips NA, Bedirian V, Charbonneau S, Whitehead V, Collin I, Cummings JL, Chertkow H (2005). The Montreal cognitive assessment, MoCA: a brief screening tool for mild cognitive impairment. J Am Geriatr Soc.

[24] He L, Lee EY, Sterling NW, Kong L, Lewis MM, Du G, Eslinger PJ, Huang X (2016). The key determinants to quality of life in Parkinson's disease patients: results from the Parkinson's disease biomarker program (PDBP).

[25] Gelfand AE, Sahu SK, Carlin BP (1993). Efficient parametrisations for normal linear mixed models. Biometrika.

[26] Rizopoulos D (2012) Joint models for longitudinal and time-to-event data with applications in R. In: Chapman & Hall/CRC biostatistics series Boca Raton: Chapman & Hall/CRC press.

[27] Mentre F, Escolano S (2006). Prediction discrepancies for the evaluation of nonlinear mixed-effects models. J Pharmacokinet Pharmacodyn.

[28] Guangyi M, Yujun S, Hao X, de-Miguel S (2015). A mixed-effects model with different strategies for modeling volume in Cunninghamia Lanceolata plantations. PLoS One.

[29] DeLong ER, DeLong DM, Clarke-Pearson DL (1988). Comparing the areas under two or more correlated receiver operating characteristic curves: a nonparametric approach. Biometrics.

[30] Wood BH, Bilclough JA, Bowron A, Walker RW (2002). Incidence and prediction of falls in Parkinson's disease: a prospective multidisciplinary study. J Neurol Neurosurg Psychiatry.

[31] Harrison MB, Wylie SA, Frysinger RC, Patrie JT, Huss DS, Currie LJ, Wooten GF (2009). UPDRS activity of daily living score as a marker of Parkinson's disease progression. Mov Disord.

[32] Kadastik-Eerme L, Muldmaa M, Lilles S, Rosenthal M, Taba N, Taba P. Nonmotor Features in Parkinson’s Disease: What Are the Most Important Associated Factors? Parkinson's Disease. 2016;2016. 10.1155/2016/4370674 PMC4853954 27195172

[34] Green J, McDonald WM, Vitek JL, Evatt M, Freeman A, Haber M, Bakay RA, Triche S, Sirockman B, DeLong MR (2002). Cognitive impairments in advanced PD without dementia. Neurology.

[33] Murakami H, Owan Y, Mori Y, Fujita K, Futamura A, Sugimoto A, Kobayakawa M, Kezuka M, Midorikawa A, Kawamura M (2013). Correlation between motor and cognitive functions in the progressive course of Parkinson's disease. Neurology and Clinical Neuroscience.

[35] Riggeal BD, Crucian GP, Seignourel P, Jacobson CE, Okun MS, Rodriguez R, Fernandez HH (2007). Cognitive decline tracks motor progression and not disease duration in Parkinson patients. Neuropsychiatr Dis Treat.

[36] Williams LN, Seignourel P, Crucian GP, Okun MS, Rodriguez RL, Skidmore FM, Foster PS, Jacobson CE, Romrell J, Bowers D, Fernandez HH (2007). Laterality, region, and type of motor dysfunction correlate with cognitive impairment in Parkinson's disease. Mov Disord.

